# Is There a Preferred Mode of Exercise for Cognition Enhancement in Older Age?—A Narrative Review

**DOI:** 10.3389/fmed.2019.00057

**Published:** 2019-03-29

**Authors:** Yael Netz

**Affiliations:** The Academic College at Wingate, Netanya, Israel

**Keywords:** mode of exercise, physical training, motor training, metabolic demands, neuromuscular demands, exercise intensity, exercise complexity

## Abstract

The aim of this review is to examine the moderating effect of the mode of exercise on the exercise-cognition relationship. Is one mode of exercise more efficient in enhancing cognition than the other? For example, is aerobic exercise preferable over balance training? Based on official guidelines for old age, exercise modes include aerobic activity, strength (resistance) training, flexibility, balance, and coordination. In relation to cognition, these exercise modes are further divided into two categories: physical training—aerobic and strength, and motor training—balance, coordination, and flexibility. The physical training activities are repetitive and automatic in nature, and require high metabolic energy and relatively low neuromuscular effort. The motor activities involve high neuromuscular demands and relatively low metabolic demands. In addition, there are specific movement skills that require more neuromuscular effort (e.g., Tai Chi), and sometimes also greater metabolic demands (e.g., tennis). Selected studies examining the effect of various modes of exercise on cognition contend that both training categories affect neuroplasticity, and consequently cognitive functioning. However, there are two main differences between them: (1) Physical training affects cognition via improvement in cardiovascular fitness, whereas motor training affects cognition directly; (2) Physical training affects neuroplasticity and cognition in a global manner, while motor training is task-specific in increasing brain neuroplasticity and in affecting cognition. Examining the underpinnings of these pathways reveals that there is a difference in the underlying forces behind the two training categories. In the physical training category, it is the *intensity* of training that enhances neuroplasticity and consequently improves cognition, while in the motor activities it is the task *complexity* that increases neuroplasticity, which improves cognition. Dual-task training, which includes cognitive demands in addition to physical or motor activity, has proven more effective in improving cognitive functioning than a single task. The implications are that if all training components traditionally recommended by official bodies—physical as well as motor training—are efficient in enhancing cognition, then we merely have to emphasize the inclusion of all exercise modes in our routine exercise regimen for physical as well as cognitive health in advanced age.

## Introduction

Research on the effect of physical activity on cognition in advanced age has experienced enormous growth in the last decade, with quite a few reviews strongly indicating the important role of a physically active lifestyle in reducing cognitive decline [e.g., (1–6)]. The most prominent moderators affecting this relationship are the dose of exercise—intensity, duration, frequency; the cognitive variables assessed; and the exercise mode (5). While the first two have been comprehensively explored, the exercise mode still remainsunder-investigated.

The aim of this review is to examine the moderating effect of the mode of exercise on the exercise-cognition relationship. Is one mode of exercise more efficient in enhancing cognition than the other? For example, is aerobic exercise preferable over balance training? In order to answer this question, I will first illustrate the structure of the modes of exercise as related to cognition, followed by an examination of historical trends in the research on mode of exercise and cognition. I will then review typical selected studies examining the effect of specific modes of exercise on cognition, followed by proposing a model of the different underlying forces behind the various modes and the different paths leading from physical activity to enhanced cognition. Finally, I will examine the information on the exercise-cognition relationship, relating its application to the official recommendations for physical activity in old age.

## Modes of Exercise and Cognition—Two Main Categories

The traditional modes of exercise are comprised of assorted sets of movements aimed at enhancing various body systems. Based on the guidelines of exercise in old age recommended by official bodies, exercise modes include aerobic activity, strength training (resistance exercise), flexibility, balance, and coordination (7–9).

**Figure 1:**
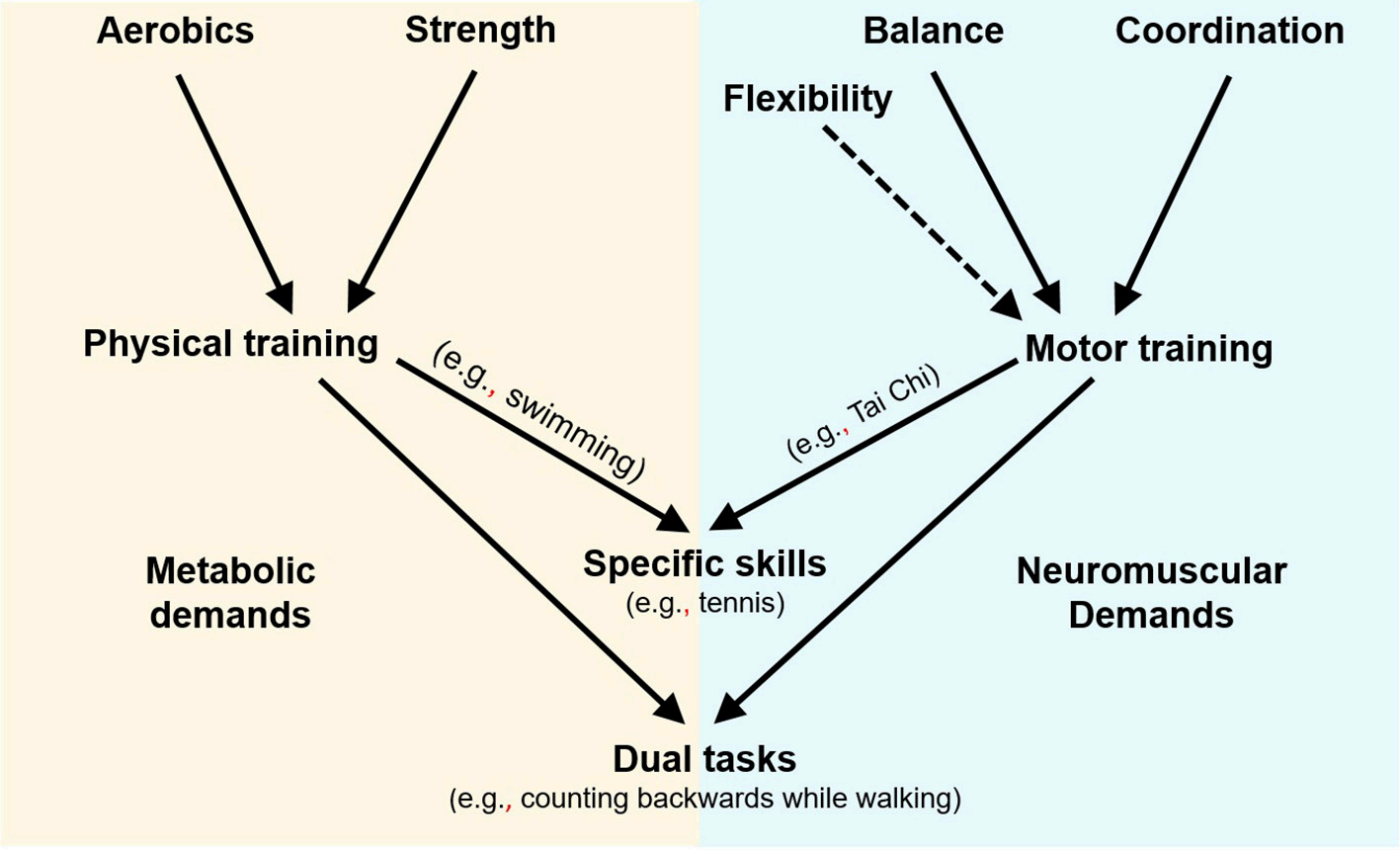
Basic modes of exercise—neuromuscular vs. metabolic demands.

In relation to cognition, these exercise modes could be further divided into two categories based on the inherent type of energy required to produce the activity (10): *physical* vs. *motor activities* (see Figure 1). The physical training activities are primarily aerobic exercise, but they also include strength training. These activities are repetitive and automatic in nature, and require high metabolic energy and relatively low neuromuscular effort (strength training requires more neuromuscular effort than aerobic exercise). The motor activities, on the other hand, consist of balance and coordination, and involve high neuromuscular demands and relatively low metabolic demands. Specifically, they require perceptual and higher-level cognitive processes, such as attention (11). Flexibility (extending the range of motion in the joints) is classified as a motor activity, although it does not entail high neuromuscular effort.

In addition to the basic motor activities, there are *specific movement skills* (Figure 1) that branch off from the two categories. For example, yoga, Tai Chi, quiet dances, and some exergames (movement-based video games) stem from the basic motor activities but generally require higher neuromuscular effort than simple balance and coordination activities. Bicycle riding and swimming, on the other hand, which are physical (metabolic) in nature, require neuromuscular effort, but only in the motor learning phase. Once the skill is well-acquired, the neuromuscular efforts are minimal. A more demanding subcategory of the specific skills group is a combination of physical and motor activity requiring both metabolic and neuromuscular energy, for example tennis, basketball, or energetic dancing and exergames.

While the various cognitive/motor/physical demands of the specific skills group are inseparable and merge into one set of movements leading to an end—that is, performing that specific skill (e.g., tennis or yoga or dancing), *dual-task activities* include a controlled combination of two tasks or activities, performed simultaneously, and arbitrarily designed as a means to promote basic motor systems such as postural control or cognitive functioning. They may include: (1). a combination of motor-cognitive tasks, such as standing on one foot and counting backwards by 3 from 100; (2) a combination of physical and cognitive activities, such as walking fast on a treadmill while performing various tasks presented on a screen; or (3) a combination of two motor tasks, such as standing on one foot and throwing/catching a ball. Most of the recent dual-task studies use exergames. These studies compare traditional activities, such as balance, to balance + cognition (exergame), or aerobics, to aerobics + cognition (exergame). The exergame includes movement (motor or physical) simultaneously performed with a cognitive stimulation.

## Mode of Exercise and Cognition—Historical Aspects

Although aerobic exercise involves few neurocognitive demands, historically it was the first exercise mode to be explored in relation to cognitive functioning. Perhaps this was because of the perception that exercise meant primarily energy expenditure, and physical fitness meant cardiovascular (aerobic) fitness (12). The emergence of this perception has roots in the “evolution of sedentarism” due to modern technology. While in ancient times there was no need for purposeful exercise, as survival forced human beings to be in motion, the overwhelming reduction in daily energy expenditure in the Twentieth-century, along with an increase in cardiovascular diseases (13), brought about the promotion of aerobic exercise as the main mode of exercise for fitness and health promotion (12, 14). Another reason may be that aerobic exercise is more measurable than other activities in terms of the intensity of exercise, thus its effect on other body systems, such as on the activity of the brain, was more quantifiable (15).

Following aerobic exercise, it was strength (resistance) training that was explored and promoted in older populations (16, 17). The importance of balance and coordination exercise evolved only later on, when life expectancy increased and the issue of falls in old age came into focus (18–20).

## Physical (Metabolic) Activities and Cognition—Selected Studies

In the aerobic exercise studies, older adults exposed to moderate intensity aerobic exercise are compared to light—mostly stretching and toning—control groups (21–25). The intensity of training is pre-determined, based on individual aerobic capacity (estimated or measured VO2 max—maximal oxygen consumption), and well-controlled during the training. Importantly, all these studies reported a significant improvement in fitness in addition to improvement in cognitive functioning, mainly executive functions.

Dustman and colleagues (21) are considered pioneers in this area. They observed improvements as a result of aerobic training in various neurocognitive functions, including recall and reproduction of verbal and auditory materials, visuo-motor speed, critical flicker fusion threshold, and mental flexibility—the ability to shift perceptual set as measured by the Stroop Interference Test. They argued that the effects of exercise were primarily on central rather than on peripheral mechanisms, and that the effect appeared to be widespread in a variety of areas.

Kramer and colleagues (22) took the research one step further. They were the first to show that the effect of aerobic exercise on neurocognitive functions is selective. It affects only executive functions supported by the frontal and prefrontal cortex and not non-executive functions. For example, aerobic exercise improved the reaction time on tasks requiring switching between tasks (executive functions), as opposed to continuing performance of the same task (non-executive functions).

New technology that enables looking into the brain has brought a new wave of studies examining the triad of brain, cognition, and exercise. Two groups of researchers—Colcombe (23, 26) and Erickson (24, 27) and their colleagues, are considered pioneers in conducting research on aerobic exercise and the brain. Both groups first conducted a correlation-type study, followed by an intervention study. Colcombe and colleagues were pioneers in exploring the relationship between aerobic fitness and brain tissue loss (26), and the effect of aerobic exercise on brain volume (23) in aging humans, using neuroimaging (magnetic resonance imaging—MRI). In their cross-sectional study (26) they found robust declines in tissue densities as a function of age in the frontal, parietal, and temporal cortices, but the losses in these areas were substantially reduced as a function of cardiovascular fitness. Carrying on this line of thought, they conducted an intervention study (23) and observed significant increases in brain volume in both the gray and white matter regions, primarily located in prefrontal and temporal cortices, as a function of fitness for the older adults who participated in the aerobic fitness training. It should be noted, however, that no neuropsychological tests were performed in this study, therefore it is not clear how these volumetric changes relate to changes in cognitive scores.

Following this line of reasoning, Erickson and colleagues conducted a correlation-type study (27) followed by an intervention study (24), but with triad measurements including aerobic activity, brain measures, and cognitive measurements. In their longitudinal correlation-type study they followed older adults for 13 years, showing that a greater amount of aerobic training (walking) predicted greater volumes of frontal, occipital, entorhinal, and hippocampal regions 9 years after assessing the aerobic activity. Furthermore, greater gray matter volume with aerobic activity cut the risk for cognitive impairment in half at 13 years after the baseline assessment of the aerobic activity. In their intervention study (24), Erickson and colleagues showed that aerobic exercise training increases the size of the anterior hippocampus, leading to improvements in spatial memory. Importantly, they also demonstrated that increased hippocampal volume was associated with greater serum levels of Brain-derived Neurotrophic Factor (BDNF), a mediator of neurogenesis in the dentate gyrus. Furthermore, while hippocampal volume declined in the control group, higher pre-intervention fitness partially attenuated the decline, suggesting that fitness protects against volume loss. The novelty of this study was that aerobic exercise training was effective at reversing hippocampal volume loss in advanced age, which was accompanied by improved memory function.

A more recent study provided additional comprehensive support for the relationship between aerobic exercise, brain structure, and cognitive performance (25). The researchers of this study used a comprehensive neuropsychological test battery in which cognitive constructs were measured using several different tests. In addition, they assessed cortical thickness in frontal regions, as well as hippocampus volume. In this sense, this study may serve as a summary of the effect of aerobic exercise on cognition and on the brain. Results showed that aerobic exercisers, compared to controls, exhibited a broad rather than specific improvement in cognition, as indexed by a higher “cognitive score,”—a composite including episodic memory, processing speed, updating, and executive function tasks. No group differences were detected on cortical thickness, but aerobic fitness at baseline was related to greater thickness in the dorsolateral prefrontal cortex (dlPFC), and hippocampus volume was positively associated with increased aerobic fitness over time. Furthermore, changes in the “cognitive score” and dlPFC thickness were associated over time in the aerobic group only. The authors concluded that aerobic exercise had a broad influence on cognitive functioning, which may explain why studies focusing on a narrower range of functions have sometimes reported mixed results.

The link between cardiovascular fitness and cognition has also been evidenced in a wide range of cross-sectional studies that measured the association between VO2 max and cognitive tasks representing mainly executive functioning [e.g., (28–31)].

The theory behind all these aerobic exercise studies (experimental as well as cross-sectional) is that greater cardiovascular capacity (the “cardiovascular fitness” hypothesis) aids the efficiency with which oxygen and nutrients are delivered to the brain (32). Increased oxygenation provides energy for neuronal activity, which benefits cognition. More specifically, cardiovascular fitness affects cerebral blood flow (33–35), increases the density of the capillaries—cerebral angiogenesis (36, 37), causes neurotrophic stimulation (38), increases the plasticity of neurotransmitter systems (39), and increases gray or white matter volume in the prefrontal cortex (23), in the hippocampus (24), and in the cerebellum and motor cortex (40). These changes in brain structure and in neuroplasticity involve global as well as localized effects of aerobic exercise on brain and cognition (40).

Once the link between aerobic exercise and cognition was clearly established, and following a report in a meta-analysis that a combination of aerobic and strength training had a greater benefit than aerobic exercise exclusively (41), scientists and neuropsychologists moved to strength training as a potential enhancer of cognitive functioning. These studies reinforced the aerobic exercise studies reporting that strength training is also effective in altering measures of executive functions [see (42–44)].

Two leading studies explored the effect of resistance exercise on cognition—one assessed men (42) and one women (43). Cassilhas et al. (42) assessed the effect of moderate and high intensity resistance training in older men, as compared to a control group, on a few cognitive tests. Following 6 months of training, both of the experimental groups significantly improved in short- and long-term memories, in executive functioning, and in attention. Furthermore, a significant increase in insulin-like growth factor-1 (IGF-1) serum concentrations was found in both groups, which was associated with the improvement in cognitive processes.

Following the assessment of two groups of intensity in older men, a later study explored the effect of two frequencies—once and twice a week—of resistance training on cognition in a group of older women (43). After 12 months, both frequencies of resistance training demonstrated improvement in selective attention and conflict resolution (Stroop) as compared to a balance-and-toning control group. At 2-year follow-up, both frequencies performed better in executive functions as compared to the balance-and-toning control. Additionally, twice-weekly resistance training promoted memory and reduced cortical white matter atrophy relative to balance-and-toning, suggesting that resistance training may have a long-term positive impact on cognition and white matter volume in older women (45).

In another report, a month of training, twice or three times a week, was sufficient to create a significant improvement in Digits Backward and Stroop C tasks (executive functions) in favor of strength training as compared to a waiting list group (44).

It should be noted that the same mediating mechanisms were attributed to the improvement in cognition following resistance training as following aerobic exercise, for example better blood flow in the brain (42, 44) and increased neuroplasticity (44).

Interestingly, quite a few studies demonstrated improvement in executive functions following a single session of aerobic [e.g., (46, 47)] or resistance [e.g., (48)] exercise. These improvements are usually explained by energetic models such as the regulation of brain dopamine and noradrenalin, which acts to increase arousal level and leads to improved cognitive functions (49).

While aerobic exercise and strength training focus on different body systems, the common denominator between them is that they both use automatic movements affecting metabolic and energetic processes, which are based on controlled, quantifiable intensity. These processes mediate between the exercise and the cognitive functioning.

## Motor (Neuromuscular) Activities and Cogntion—Selected Studies

A shift in this line of research took place with the notion that typical balance and coordination training, such as moving with a narrow base of support, eye-hand coordination, or arm-leg coordination, relies more on neuromuscular demands than the highly automatic movements typical to aerobic or strength training. This notion inspired the idea that these movements may stimulate changes in information processing, specifically the ability to handle visual and spatial information (11). Similar to the previously mentioned pattern of development in the research on aerobic exercise and cognition (23, 24, 26, 27), Voelcker-Rehage et al. conducted a cross-sectional study (11), followed by an intervention study (50) with a triad measurement of coordination (as compared to aerobic) training, brain measures, and cognitive assessments. The functional brain imaging data of the cross-sectional data revealed that, in comparison with a control group, both physical (aerobic capacity) and motor (balance, coordination, agility, flexibility) fitness were associated with more efficient cognitive processing, as indicated by fewer cortical activation in areas responsible for executive control (superior and middle frontal cortex). However, motor fitness was also associated with greater activation of the right inferior frontal–posterior parietal network, which is involved in visuo-spatial processing and action initiation (11).

The findings of the intervention study (50) comparing the effect of 12 months of coordination training (dancing) to aerobic exercise (walking) and to a control group were as follows: (1) Both aerobic (physical) and coordination (motor) training improved executive functions, but only coordination exercise improved perceptual speed; (2) Aerobic training caused a reduction of task-related activation in several superior, middle; and medial frontal, and superior and middle temporal cortical areas, as compared to increased activation in these areas for the control group. In contrast, coordination training generated an increased activation in the inferior frontal gyrus and the superior parietal cortex, as well as in subcortical structures like the thalamus and caudate body; (3) The brain and cognitive changes in the aerobic group were associated with an improvement in cardiovascular fitness. In contrast, the cognitive and brain changes in the coordination group were not associated with changes in motor performance.

Importantly, this study was later supported by a similar study (51) comparing dance with an emphasis on balance training, to physical fitness training consisting of aerobic and strength training. Although this later study (51) did not include cognitive measurements, it supported the previous study (50) in two ways: (1) Both groups improved in brain measures but more changes were observed in the dance group. Specifically, both dancing and fitness training led to increases in hippocampal subfield volumes. However, participants of the dance group showed volume increases in more subfields of the hippocampus. (2) While only the dancers improved in balance, there was no correlation between the hippocampal volume and the improvement in balance. That is, the changes in motor performance were not associated with the brain changes.

Interestingly, another study exploring the effect of physical training vs. motor training in older adults reported similar results (52). In that study the effect of strength (resistance) training on cognition (inhibition) was compared to that of neuromuscular (coordination, balance, agility) training. The results indicated that both groups improved in both functional mobility and cognition. However, mediation analysis suggested that different mechanisms underlie the effects of neuromuscular and strength training. While neuromuscular training seemed to directly affect inhibitory capacity, strength training seemed to affect it indirectly through gains in muscular strength.

A more recent study examining the effect of Tai Chi (motor training) on cognition as compared to brisk walking (physical training) and to an age-matched sedentary control group reported similar results (53). Specifically, this study used the Stroop test for three conditions: naming, inhibition, and executive condition. Both training groups showed a shorter reaction time in executive function than the control group. However, the Tai Chi group showed a shorter reaction time in the naming and the executive conditions, and fewer mistakes in the inhibition conditions than the brisk walking. A possible explanation is that Tai Chi training involves learning and memorizing new skills and new movement patterns, as well as sustaining attention, which could be helpful for improving working memory, divided attention, and overall executive ability. Compared with brisk walking, the benefits of Tai Chi may be attributed to the increased cognitive demands of practicing the exercise.

Notably, Tai Chi belongs to the mindful activities that have recently become popular among older adults, and there is a growing line of research relating these activities to cognition. Yoga is one of them (54), but more so Tai Chi. A meta-analysis on Tai Chi found that it enhances cognitive function in older adults, particularly executive functioning (55). It was suggested that Tai Chi trains agility and mobility, which impacts cognitive function via neurophysiological pathways unique to those associated with aerobic exercise. However, while aerobic fitness is associated mainly with executive functioning, mobility and agility are associated with both executive functioning and perceptual speed. Another suggested mediating mechanism was that, like learning dancing and juggling which are known to foster brain changes, Tai Chi involves learning and memorization of new skills and movement patterns. Tai Chi also includes training in sustained attentional focus, shifting, and multi-tasking, which could help train working memory, divided attention, cognitive flexibility, and overall executive function. An additional mediating mechanism is the meditative and relaxation power of Tai Chi to reduce anxiety and depression, which may impact cortisol and other stress-related pathways of cognitive decline.

In conclusion, studies comparing the cognitive benefits of motor training to those of physical training argue that both are beneficial, but motor training may provide additional benefits that are not achieved in physical training.

## Physical (Metabolic) vs. Motor (Neuromuscular) Activities and Cognition—Different Driving Mechanisms

**Figure 2:**
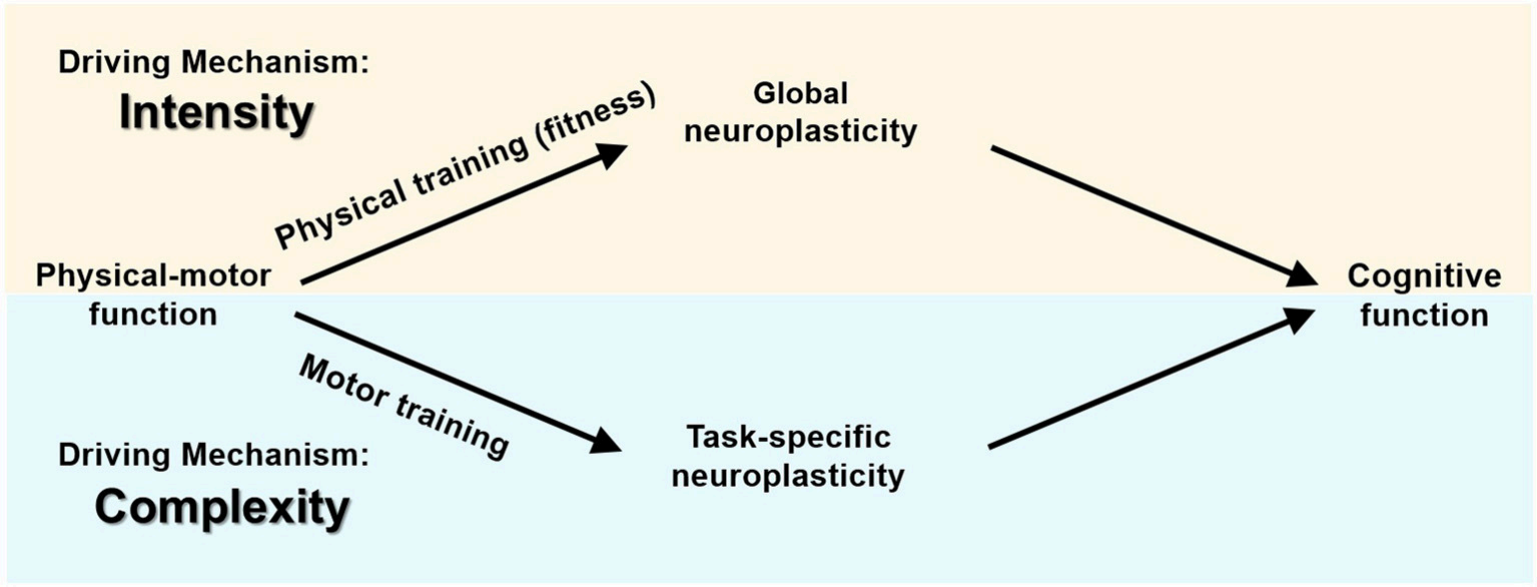
Physical-motor training and cognition—different pathways and driving mechanisms.

The pattern of the results of all the above mentioned studies implies two mechanisms for physical activity affecting cognitive functions via two different pathways (Figure 2). Both training categories affect neuroplasticity and consequently cognitive functioning. However, there are two main differences: (1) Physical training affects cognition via improvement in cardiovascular fitness, whereas motor training affects cognition directly; (2) Physical training affects neuroplasticity and cognition in a global manner, while motor training is task-specific in increasing brain neuroplasticity and in affecting cognition.

Perhaps an indirect source of support for these different mechanisms may come from studies reporting a reverse cause-effect relationship between movement and cognition. The assumption of these studies is that if motor training affects cognition directly, it is also possible that cognitive training affects motor capacity. For example, one study showed that dual-task cognitive training significantly improved body sway during some static and dynamic balance tasks (56). A meta-analysis summarizing these studies concluded that cognitive training interventions can improve mobility-related outcomes, especially during challenging walking conditions requiring higher-order executive functions (57). On the other hand, it is not expected that improved cognition will improve cardiovascular capacity or muscle strength, which are the mediating mechanisms between physical training and cognition.

Examining the underpinnings of these pathways reveals that there is a difference in the underlying forces behind the two training categories in altering neuroplasticity and cognition (Figure 2). Based on previous work (15, 58), it is the *intensity* of training that enhances neuroplasticity and consequently improves cognition as a result of the physical training category, but it is the motor *complexity* (neurocognitive demands) that affects the relationship between exercise and cognition in motor training. More specifically, aerobic and strength training, consisting of automatic repetitive movements, affects metabolic and energetic processes that alter neuroplasticity and cognition. The driving force behind this process is intensity. A certain intensity, expressed in level of cardiorespiratory or muscular effort and performed at a certain frequency, duration etc., is required to initiate the route (pathway) of training-neuroplasticity-cognition. In contrast, it is the task complexity that drives this chain of balance or coordination training affecting neuroplasticity and cognition. Contrariwise, task complexity is hardly measureable in terms of quantity, thus the dose-response effect of motor activities on cognition is difficult to determine.

## Single-Task Motor or Physical Training vs. Dual-Task Training and Cognition

**Figure 3:**
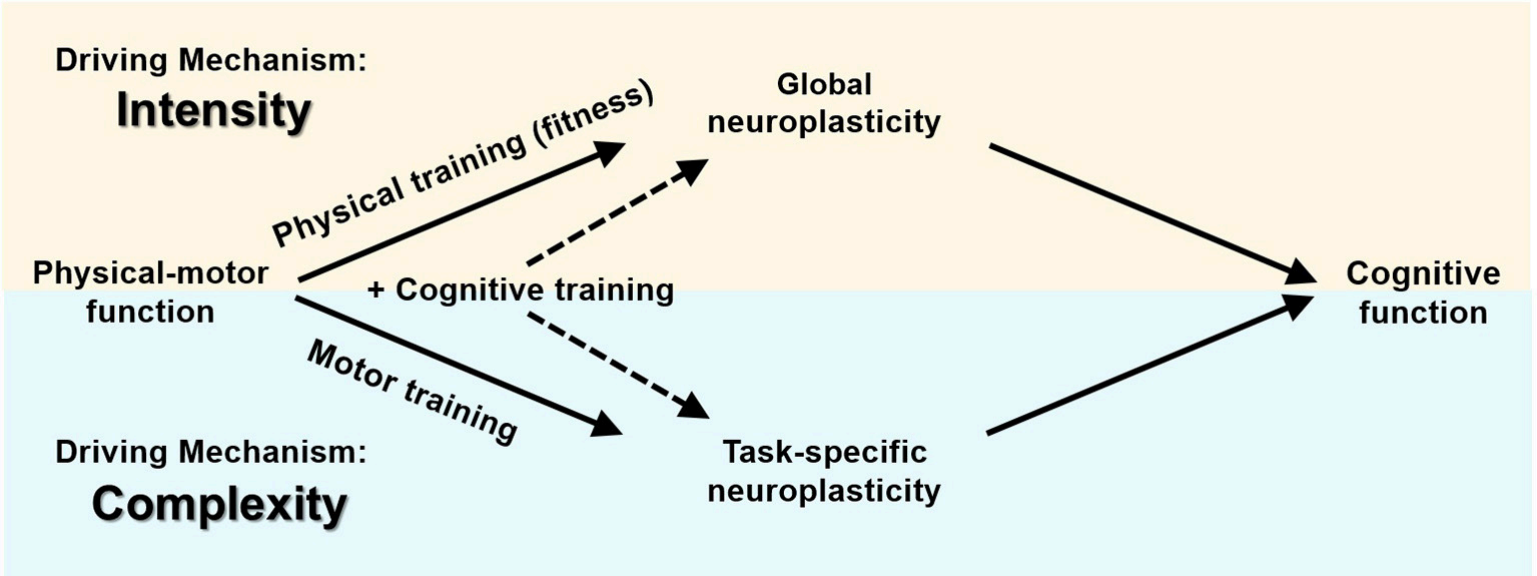
Physical-motor training and cognition—dual tasks.

One way to control and quantify complexity is to perform dual-task activities. Dual-tasking refers to the performance of two activities simultaneously (59). Dual-task training, which includes cognitive demands in addition to physical or motor activity (see Figure 3), has proven to be more effective in improving cognitive functioning than a single task (4, 60).

In real-life situations, where more attentional resources are needed or where attentional capacities are limited, as in old age, even seemingly simple motor skills may become problematic when performed simultaneously with an attention demanding task (61). The exceeded attentional demands may lead to interference between the two tasks, manifested in a decreased performance in one or both tasks (61). On the other hand, based on the principle that training a system means burdening it, it is recommended to experience this interference in controlled conditions, and thus attenuate the decrease in performance in real life situations. Indeed, there is consistent evidence that performing physical/motor and cognitive training simultaneously has the potential to maintain or improve cognitive efficiency during aging more than a single task (62, 63).

A review study (64) explored the influence of sequential (successive) vs. simultaneous (concurrent) dual-task exercise training on cognitive function in older adults. While the findings on sequential dual-task training as compared to cognitive or exercise training alone were inconsistent, the simultaneous dual-task interventions significantly improved cognition as compared to single-task activity, in both healthy older and clinical populations.

Following are two studies indicating the effect of simultaneous dual-task activity as compared to single task activity. One study (65) compared physical exercise alone to a physical plus mental challenge as combined in an exergame (interactive game). Specifically, this study compared the effect of a stationary ergometer with exergaming (cyber-cycle) to that of a stationary ergometer only. Participants in the cyber-cycle and stationary ergometer (control condition) rode identical recumbent stationary bikes, except for the virtual reality display attached to the cyber-cycle. The outcome measures were executive functions (trail color difference, Stroop C, digit backwards), clinical status, and plasma BDNF. The cyber-cycling older adults achieved better functioning than the traditional exercisers for the same effort in all measures.

Interestingly, similar results were yielded in a study exploring motor (balance) exercise alone as compared to motor exercise and exergaming (66). The exergame group performed cognitive-motor training, including an interactive video game-based physical/motor exercise. The games specifically trained executive functions—inhibition and switching, working memory, and selective attention. Participants in the balance group performed conventional balance training—repetitive static and dynamic exercises on stable and unstable surfaces to challenge their balance. The results of the brain activity measurement showed that theta relative power, assessed by electro-encephalographic (EEG) frequencies over the prefrontal cortex, significantly decreased post-intervention in favor of the exergame group. Comparing the performance in executive functions—working memory, divided attention, go/no-go, and set-shifting—indicated that while the balance group improved in set-shifting only, the exergame group demonstrated improvement in all tasks.

Anderson-Hanley and colleagues (65) provided two possible explanations for the cognitive benefits of a physical/motor + cognitive task as compared to physical/motor only: one explanation—the compound effect—was that the effect is directly due to the added mental exercise required by the exergame (cognitive training improves the targeted ability). Another explanation—the synergistic effect—was that the effect is due to the interactive nature of combined physical and cognitive exercise. This implies a transfer of improvement to tasks that have not been explicitly trained.

Support for this synergistic effect explanation is provided by another study (67). In that study, older adults were trained with a simultaneous verbal working memory and cardiovascular treadmill training, which was compared to single working memory training and a passive control group. While both training groups significantly improved their performance in the executive control task as compared to the passive control group, only the simultaneous training group demonstrated a significant increase in the paired-associates learning task when compared to the cognitive-only training group. Even though the mean training progress did not differ between the two training groups, the performance variance of the cognitive-only training group tended to be large while the training performance in the simultaneous-training group was more homogeneous. The explanation was that the physical activation could have helped in maintaining concentration and attention during training, whereas individual differences could have become more evident during cognitive-only training. In addition, the cognitive-only training condition could have been perceived as more exhausting and less interesting, and thus less motivating. In that case, the training potential of the cognitive-only training actually would be greater—at least for those who are motivated or able to keep focused, whereas the simultaneous physical activity is able to enhance motivation and promotes improvement in those who lack concentration or are exhausted after a certain time.

## Summary and Conclusions

The history of movement has faced a crisis relationship between two elemental human phenomena: On one hand there is the principle of economy—the human aspiration to reduce physical and mental efforts to a minimum, and on the other hand there is the adaptation principle—the natural dependence of human beings on movement (68). The principle of economy has led to the development of cutting-edge technology, which has “gone too far” in reducing physical and mental efforts ignoring the dependence of human beings in physical and mental activities. While in the past there was no need for purposeful exercise, as survival forced human beings to be in motion, the overwhelming reduction in daily energy expenditure in the Twentieth-century, along with the increase in chronic diseases caused by this movement reduction, has generated the promotion of purposeful exercise as a requirement for survival and health. Specifically, purposeful exercise is comprised of assorted sets of movements aimed at enhancing various body systems.

The objective of this review was to examine whether one mode of purposeful exercise is more efficient than another in enhancing cognition in advanced age. The exercise modes were divided into two categories based on the inherent type of energy required to produce the activity: *physical* vs. *motor* activities. In this review I contend that both training categories affect neuroplasticity and consequently cognitive functioning. However, there are two main differences: (1) Physical training affects cognition via improvement in cardiovascular fitness, whereas motor training affects cognition directly; (2) Physical training affects neuroplasticity and cognition in a global manner, while motor training is task-specific in increasing brain neuroplasticity and in affecting cognition. In addition, examining the underlying forces behind the two training categories in altering neuroplasticity and cognition reveals that in physical training it is the *intensity* of training that enhances neuroplasticity and consequently improves cognition, whereas in motor training it is the motor *complexity* that affects the relationship between exercise and cognition. While intensity is measureable, complexity is hardly measurable, and thus the dose-response effect of motor activities on cognition is difficult to determine.

One way to control and quantify *complexity* is to perform dual-task activities. *Dual-task activities* include a controlled combination of two tasks or activities, performed simultaneously, and are arbitrarily designed as a means to promote basic motor systems such as postural control or cognitive functioning. Dual-task training that includes cognitive demands in addition to physical or motor activity has proven more effective in preserving or improving cognitive functioning than a single task.

It should be noted that this review is limited in that it is not a systematic review. It is more of an historical examination of the development of the research concerning the effect of exercise on cognition. It is based on highly cited studies—some of these are considered milestones in the development of the research on the effect of exercise on cognition. For example, Dustman and colleagues (21), cited 689 times, are considered pioneers in exploring the effect of exercise on cognition. Kramer and colleagues (22), cited 1,556 times, broke new ground in showing that the effect of aerobic exercise on cognition is selective—it affects mainly executive functions. Colcombe et al. (23, 26), as well as Erickson and colleagues (24, 27), are considered pioneers in conducting research on aerobic exercise and the brain. The first group (23, 26) elaborated on enhanced gray and white matters as related to aerobic exercise, while the second group (24, 27) concentrated on increased hippocampal volume and improved memory as related to aerobic exercise. One of these studies (23) was cited 1,704 times, and another (24)−2,608 times. Research on the effect of motor activities on cognition was published later on. A study was reviewed that is considered a milestone in this area—comparing coordination activities to aerobic activity, indicating the specific advantages of coordination activities in addition to the aerobics (50). This study was cited 260 times.

It is possible that a systematic review may produce different conclusions than the conclusions drawn in this review. On the other hand, this review emphasized the fact that most studies examining the effect of exercise on cognition concentrate on metabolic activities (aerobic and strength), and only in the last few years have additional studies examined the neuromuscular activities (balance and coordination) as potential enhancers of cognition. Clearly, further studies are needed for assessing the effect of motor (neuromuscular) activities on cognition. Complexity is the driving mechanism attributed to motor activity in relation to cognition. Further studies are needed for assessing the dose-response relationship between level of complexity and cognition.

How does the information on the exercise-cognition relationship affect the official recommendations for physical activity in old age? Fortunately, the implications are that if all training components traditionally recommended by official bodies—*physical as well as motor training*—are efficient in enhancing cognition, then we merely have to emphasize the inclusion of all exercise modes in our routine exercise regimen for physical as well as cognitive health. It is also recommended that more cognitive stimulations, such as dual-task activities involving both a movement-base as well as cognitive tasks, be implemented in the exercise routine.

Interestingly, the same technology that reduced physical and mental efforts to a minimum (i.e., automobiles, house appliances, GPSs, etc.) is now being used to stimulate physical and mental energy expenditure (i.e., exergames). If all modes of exercise are related to cognition via various physical, biochemical, or neurological mediating mechanisms, as well as various driving forces, is it not an indication of the wholeness of the human being, and of the need to investigate human movement as it relates to all aspects of life?

## Author Contributions

The author confirms being the sole contributor of this work and has approved it for publication.

### Conflict of Interest Statement

The author declares that the research was conducted in the absence of any commercial or financial relationships that could be construed as a potential conflict of interest.
